# The first Danish family reported with an AQP5 mutation presenting diffuse non-epidermolytic palmoplantar keratoderma of Bothnian type, hyperhidrosis and frequent Corynebacterium infections: a case report

**DOI:** 10.1186/s12895-016-0044-3

**Published:** 2016-06-03

**Authors:** Anne Bruun Krøigård, Liv Eline Hetland, Ole Clemmensen, Diana C. Blaydon, Jens Michael Hertz, Anette Bygum

**Affiliations:** Department of Clinical Genetics, Odense University Hospital, Odense, Denmark; Department of Dermatology and Allergy Centre, Odense University Hospital, Odense, Denmark; Department of Clinical Pathology, Odense University Hospital, Odense, Denmark; Centre for Cell Biology and Cutaneous Research, Blizard Institute, Bart and the London School of Medicine and Dentistry, Queen Mary University of London, London, UK

**Keywords:** Diffuse non-epidermolytic palmoplantar keratoderma, Palmoplantar keratoderma of Bothnian type, Corynebacterium infection, *AQP5* gene, Aquaporin 5, “Hand-in-the-bucket-sign”, Aquagenic wrinkling

## Abstract

**Background:**

An autosomal dominant form of diffuse non-epidermolytic palmoplantar keratoderma, palmoplantar keratoderma of Bothnian type, is caused by mutations in the *AQP5* gene encoding the cell-membrane water channel protein aquaporin 5 leading to defective epidermal-water-barrier function in the epidermis of the palms and soles.

**Case presentation:**

We report the first Danish family diagnosed with diffuse non-epidermolytic palmoplantar keratoderma of Bothnian type in which fourteen individuals are potentially affected. The proband, a 36-year-old male had since childhood been affected by pronounced hyperhidrosis of the palms and soles along with palmoplantar keratoderma. He reported a very distinctive feature of the disorder, aquagenic wrinkling, as he developed pronounced maceration of the skin with translucent white papules and a spongy appearance following exposure to water. The patient presented recurrent fungal infections, a wellknown feature of the condition, but also periodic worsening with pitted keratolysis and malodour due to bacterial infections.

**Conclusions:**

Palmoplantar keratoderma of Bothnian type, which may be associated with hyperhidrosis, is frequently complicated by fungal infections and may be complicated by Corynebacterium infections.

**Electronic supplementary material:**

The online version of this article (doi:10.1186/s12895-016-0044-3) contains supplementary material, which is available to authorized users.

## Background

Palmoplantar keratodermas (PPKs) comprise a clinically and genetically heterogenous group of hereditary disorders of the skin characterized by thickening of the stratum corneum of the palms and soles. In some cases the skin lesions are accompanied by associated diseases [1]. Based on the clinical presentation, PPKs are divided into four subtypes, including diffuse, punctate, focal and striate PPK [2]. Diffuse PPK can be further subdivided histopathologically into epidermolytic and non-epidermolytic forms depending on the presence or absence of cytolysis in the upper spinous and granular layers of the epidermis. Diffuse non-epidermolytic PPK of Bothnian type (PPKB), is not accompanied by associated diseases and was initially described in two Swedish families [3]. PPKB (OMIM 600231) is autosomal dominantly inherited and initial studies linked the underlying gene defect to chromosome 12q11-q13 [3, 4]. In 2013, the genetic cause of PPKB was established as due to mutations in the *AQP5* gene, located at 12q13.12 [5]. In the northernmost region of Sweden the condition is relatively common with a reported prevalence of 0.3−0.55 % [3].

The clinical presentation of PPKB include diffuse palmoplantar hyperkeratosis with a yellowish tint over the whole of the palms and soles and acral hyperhidrosis [5]. The symptoms usually presents in early childhood, in some cases as young as three months of age [6]. Secondary dermatophyte infections are frequent. A distinctive feature of this type of PPK is a whitish spongy appearance of the skin following exposure to water. The phenotypic apperarence of PPKB varies and in some individuals, clinical signs of the condition are only seen after exposure to water [5].

In this study, we present the first Danish family with autosomal dominant PPKB, caused by a heterozygous p.Arg188Cys mutation in the *AQP5* gene. The proband presented with hyperhidrosis and subsequent complications caused by superinfections with dermatophytes and Corynebacteria.

## Case Presentation

**Fig. 1:**
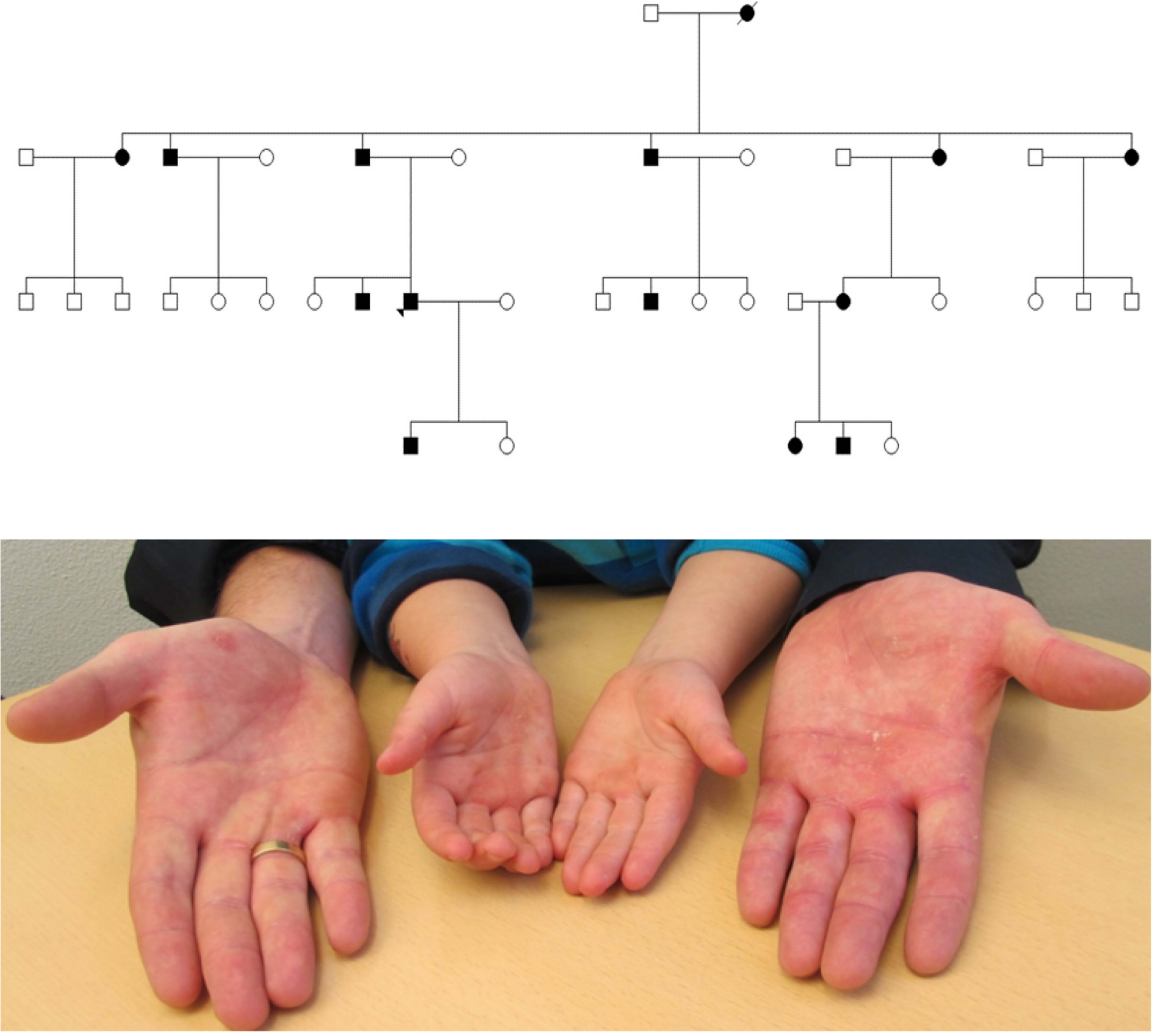
*Upper panel*: Family pedigree of the family affected by diffuse non-epidermolytic palmoplantar keratoderma which is autosomal dominantly inherited. Family members affected by the condition are marked in black. The proband is indicated by the black triangle. *Lower panel*: A similar phenotypic appearance were present in all family members. Here, exemplified by the 36-year old male proband and his eight-year old son

**Fig. 2:**
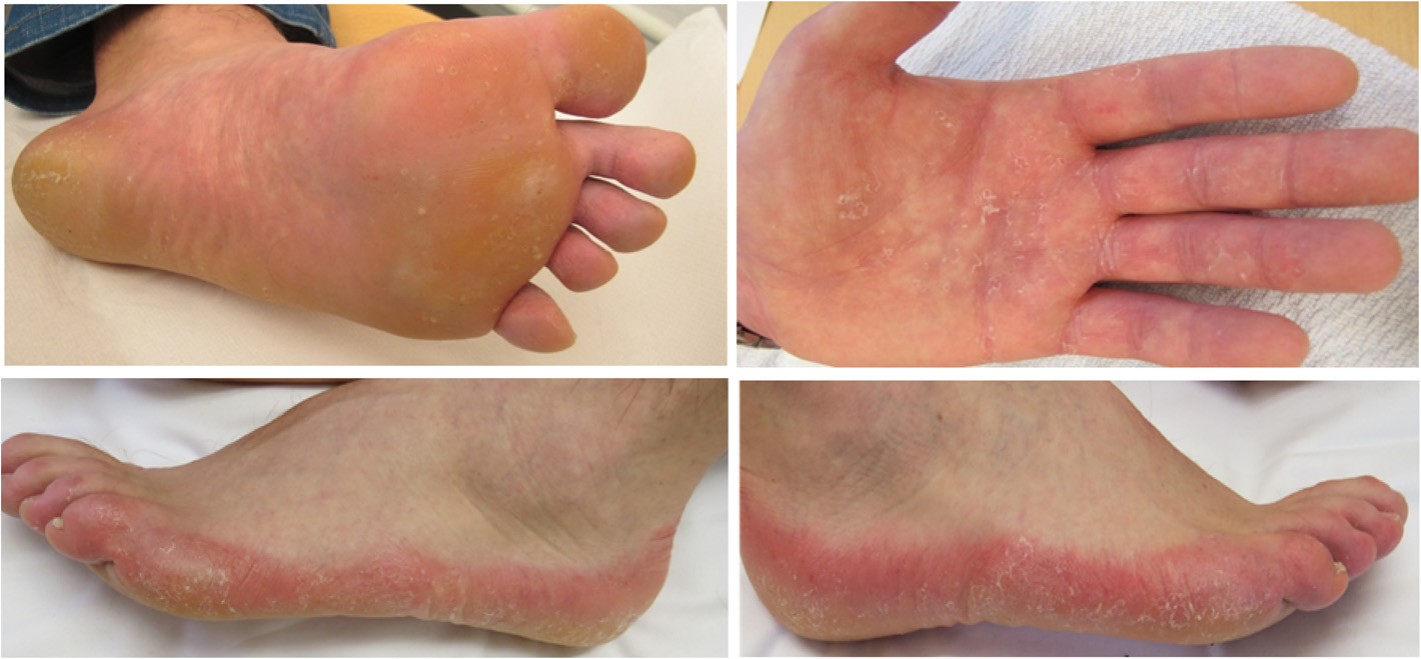
Phenotypic appearance of the 36-year old male proband affected by diffuse nonepidermolytic palmoplantar keratoderma. The palmar and plantar skin was affected by yellow tinted keratoderma, pitted keratolysis and erythrokeratotic plaques with a clear demarcation to normal skin on the dorsum of the hands and feet. Sparing of the skin of the arches were seen on planta pedis

A large four-generation Caucasian family was ascertained, including 14 affected individuals, as seen in Fig. 1. The proband was a 36-year-old male, affected since childhood by palmoplantar keratoderma, pronounced hyperhidrosis and recurrent secondary dermatophyte infections on affected skin. Examination of the skin revealed a mild, yellow tinted diffuse keratoderma of palms and soles, pitted keratolysis and erythematous keratotic plaques with a clear demarcation on the margins of the hands and feet, as seen in Fig. 2. Sparing of the skin was seen in the arches of the planta pedis. Physical examination was non-contributory. The skin lesions were periodically foul-smelling and the patient suffered from recurrent tinea pedis and onychomycosis caused by dermatophyte infections. Thirteen additional family members presented with a similar phenotype. Initially, the diagnosis epidermolytic palmoplantar keratoderma was suspected. Mutational screening of *KRT1*, *KRT9* and *KRT16* genes provided negative results.

**Fig. 3:**
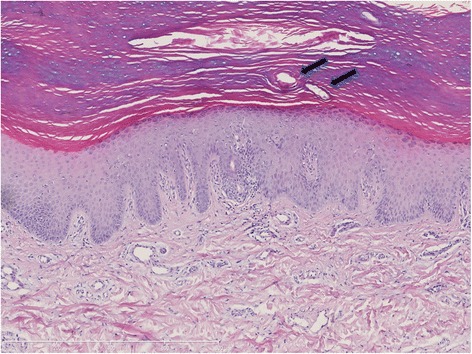
Punch biopsy from the foot, hematoxylin and eosin stained. The stratum corneum is thickened with epidermal acanthosis. The acrosyringeal intracorneal ducts are dilated (*arrows*), and focally miliaria rubra like changes with spongiosis of the eccrine duct and lymphocytic infiltration around the duct is seen

The biopsy showed a markedly thickened stratum corneum with a prominent stratum granulosum and a moderate acanthosis, as seen in Fig. 3. The acrosyringial ducts were remarkably dilated as well in the epidermis as in the stratum corneum. The distal part of the eccrine ducts in the papillary dermis, however, were without recognizable dilatation. Focally, changes like those seen in miliaria rubra with spongiosis and exocytosis of lymphocytes around the intraepidermal acrosyringial channels were noted. The lymphocytic infiltration extended into the papillary dermis around and into the distal parts of the dermal sweat ducts, but the hyperkeratotic plugging, often seen in miliaria rubra above the spongiosis, was not present in this biopsy. The proximal part of the sweat ducts and the secretory coil appeared normal. Spores and hyphae indicating a dermatophytosis were demonstrated in the stratum corneum. Corynebacteria, however, were not seen.

**Fig. 4:**
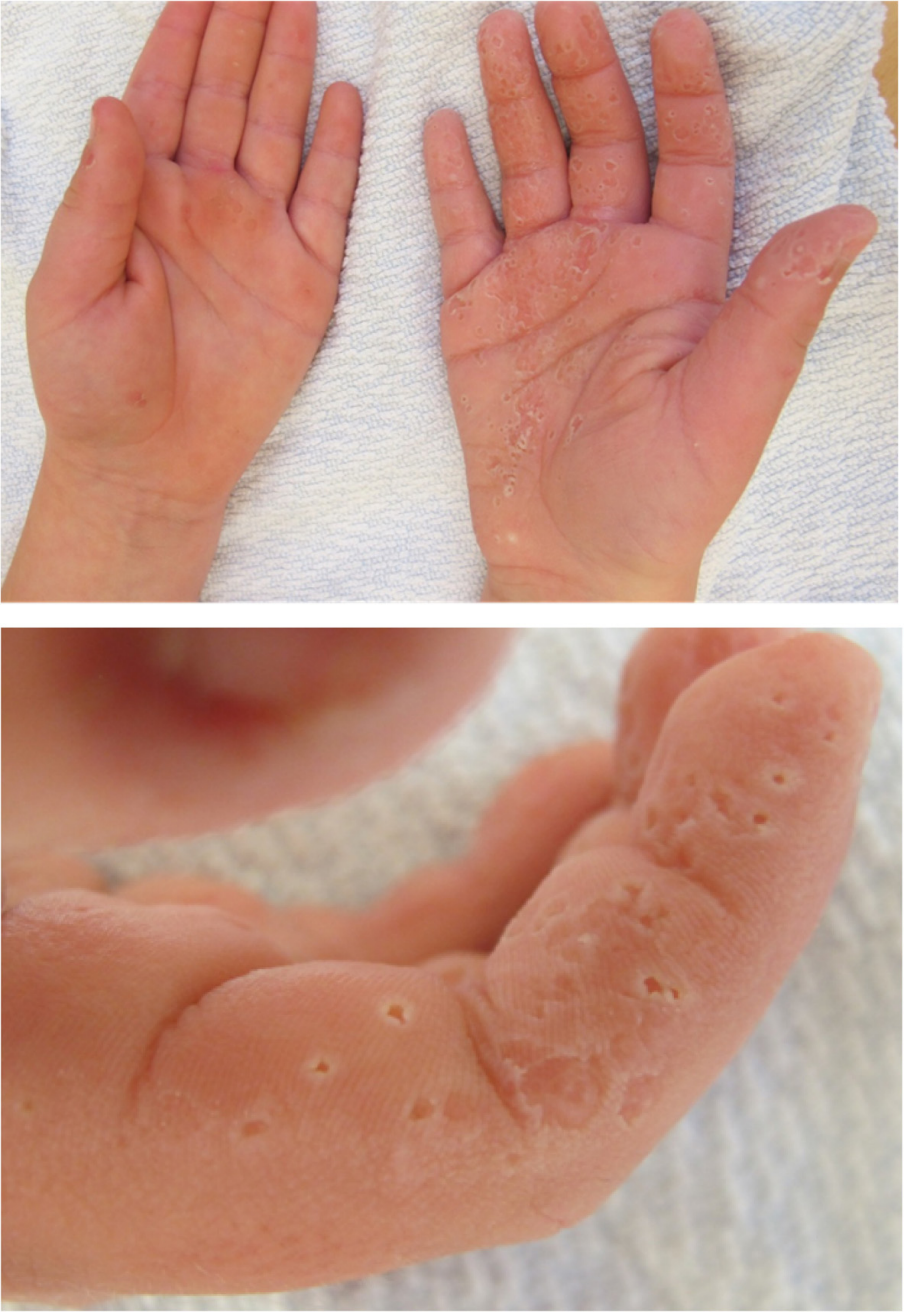
Aquagenic wrinkling, also known as “hand-in-the-bucket-sign” revealed by water immersion test. *Upper panel*: Palmar phenotype after water immersion test of only the right hand. After three minutes in water, the palmar skin on the right hand was clearly affected by translucent white papules and a whitish spongy appearance, compared to the left side which had not been exposed to water. *Lower panel*: Spongy appearance on affected skin due to swelling of the stratum corneum after three minutes exposure to water

Water immersion test revealed aquagenic wrinkling, also known as “hand-in-the-bucket-sign”, as translucent white papules and a whitish spongy appearance due to swelling of the stratum corneum was observed after three minutes exposure to water, as seen in Fig. 4. The aquagenic wrinkling lead to suspicion of transient reactive papulotranslucent acrokeratoderma [7] or aquagenic syringeal acrokeratoderma [8] but the proper diagnosis was not established until the report by Blaydon et al. [5] raised the suspicion of PPKB.

### Genetics

Following informed consent, genomic DNA purified from a blood sample from the proband was analyzed using bidirectional Sanger sequencing of the *AQP5* gene. Genetic test results available in Additional file 1: Figure S1. The patient was heterozygous for a missense mutation in the *AQP5* gene, c.562C>T, (p.Arg188Cys). This exact mutation is previously described to cause autosomal dominant PPKB [5]. The probands eight year old son was found to be heterozygous for the same *AQP5* mutation. The remaining affected family members have not consented to genetic testing.

## Superinfection with corynebacterium

**Fig. 5:**
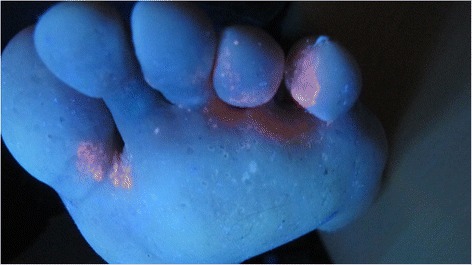
Examination of the plantar skin in Woods light showed massive coral red fluorescence in interdigital areas representing a superinfection with Corynebacterium

Interestingly, the dermatophyte infection of the plantar skin was complicated by a superinfection with Corynebacterium. Examination in Woods light showed massive coral red fluorescence in interdigital areas of planta pedis, as seen in Fig. 5. The Corynebacterium infection was treated with clindamycin and chlorhexidine with satisfactory effect.

## Discussion

We present the first Danish family diagnosed with autosomal dominant PPKB resulting from c.562C>T, p.Arg188Cys in the *AQP5* gene. According to the Human Gene Mutation Database a total of six missense mutations in the *AQP5* gene associated with PPKB have been reported to this date [9] in families of Swedish, British, Scottish and Han Chinese descent [2, 5]. The condition has also been reported in a patient from India [10].

The *AQP5* gene encodes aquaporin 5, a cell-membrane protein that allows osmotic movement of water across the cell membrane independently of solute transport [11]. Aquaporin 5 is situated in the apical plasma membrane of cells of the sweat glands, salivary glands, lacrimal gland, lung, cornea [11] and in the palmoplantar epidermis [5]. The study by Blaydon et al. reported that the expression of AQP5 in the stratum granulosum was not affected by mutations in the gene [5]. Hence, at the genetic level, mutations associated with PPKB most likely induce increased expression of the gene or excert a gain-of-function effect leading to accelerated keratinocyte water uptake. This likely explains the swelling of the stratum corneum of the epidermis following water exposure.

The notion of increased function of aquaporin 5 in PPKB is supported by the fact that immunolocalization experiments involving Sjögrens syndrome, which is characterized by hypohidrosis, revealed reduced expression of AQP5 in sweat glands from patients with Sjögrens compared to normal skin [12]. Thus, the hypohidrosis observed in Sjögrens syndrome may result from reduced expression of AQP5 and correspondingly, the hyperhidrosis of PPKB may result from an increased function of the gene product. From a clinical perspective, the hyperhidrosis characterizing PPKB entails notable discomfort to the patient and most likely is a contributing factor to the recurrent Corynebacterium infections. Conversely, it has recently been hypothesized that hyperhidrosis may be exacerbated by bacterial infection [13].

The histopathologic unspecific features of hyperkeratosis and acanthosis are in agreement with the findings in PPKB. In addition, however, we found focal changes around the intraepidermal sweat ducts as seen in conditions like miliaria rubra, also known as heat rash. To our knowledge, this has not been described in PPKB. It is possible that the biopsy was taken during an episode of hyperhidrosis, but the inflammatory changes of miliaria rubra typically do not accompany ordinary hyperhidrosis. It seems likely that the changes of stratum corneum in PPKB may obstruct the sweating and induce the miliaria rubra like changes. It certainly would be of interest to study biopsies from larger series of PPKB to assess the specificity of this feature.

Whether the pitted keratolysis results from the palmoplantar keratoderma, the hyperhidrosis or the Corynebacterium infection is not established. Treatment options for PPKB include salicylic acid 4−6 % in petrolatum, urea- or salicylic acid-containing creams and lotions and avoidance of too much moisture. The hyperhidrosis may respond to aluminum chloride-containing products or other therapeutic options of hyperhidrosis. Treatment of secondary dermatophyte infections and superinfections with bacteria like Corynebacterium is of extreme importance for optimal patient care.

The “hand-in-the-bucket-sign” is not pathonomonic of PPKB as aquagenic wrinkling of the palms following exposure to water is observed in several conditions including transient reactive papulotranslucent acrokeratoderma and aquagenic syringeal acrokeratoderma and is suggested to be more frequent than previously assumed [14]. In accordance with the PPKB genotype-phenotype correlation, aberrant expression of aquaporin 5 has been reported to be associated with aquagenic wrinkling of the palms [15]. Conversely, aquagenic keratoderma has also been suggested to be associated with mutations in the *CFTR* gene involved in cystic fibrosis [16, 17].

## Conclusions

Though increasing evidence suggests a gain-of-function effect of *AQP5* mutations related to PPKB, the exact mechanisms by which mutations in *AQP5* lead to defective epidermal-water-barrier function remains to be elucidated. Conclusively, we have yet to arrive at a final understanding and characterization of conditions of disturbed water permeability of the palmoplantar epidermal cells.

The present manuscript adds a Danish family with PPKB to the literature and emphasizes that Corynebacterium superinfections may complicate the condition.

## Abbreviations

PPK, Palmoplantar keratoderma; PPKB, Diffuse non-epidermolytic PPK of Bothnian type

## Additional file

Additional file 1:
**Figure S1.** Genetic test results of the proband. Bidirectional Sanger sequencing of the *AQP5* gene. (PDF 617 kb)
