# Supplementary material for: The first Danish family reported with an AQP5 mutation presenting diffuse non-epidermolytic palmoplantar keratoderma of Bothnian type, hyperhidrosis and frequent Corynebacterium infections: a case report
Source: BMC Dermatol. 2016 Jun 3;16:7. doi: 10.1186/s12895-016-0044-3 (PMC4891830; doi:10.1186/s12895-016-0044-3)
Supplement: Additional file 1: — Figure S1. Genetic test results of the proband. Bidirectional Sanger sequencing of the AQP5 gene. (PDF 617 kb) [file 12895_2016_44_MOESM1_ESM.pdf]

The first Danish family reported with an AQP5 mutation presenting diffuse non-epidermolytic palmoplantar keratoderma of Bothnian type, hyperhidrosis and frequent Corynebacterium infections: a case report

Additional file 1

Figure S1. Genetic test results of the proband. Bidirectional Sanger sequencing of the *AQP5* gene.

Sequence trace showing C to T substitution; the upper trace shows the normal sequence and the lower trace is from the patient:

Control

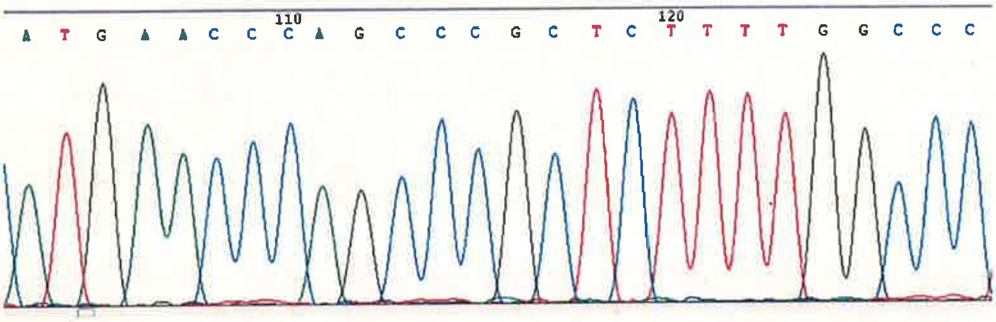

Patient

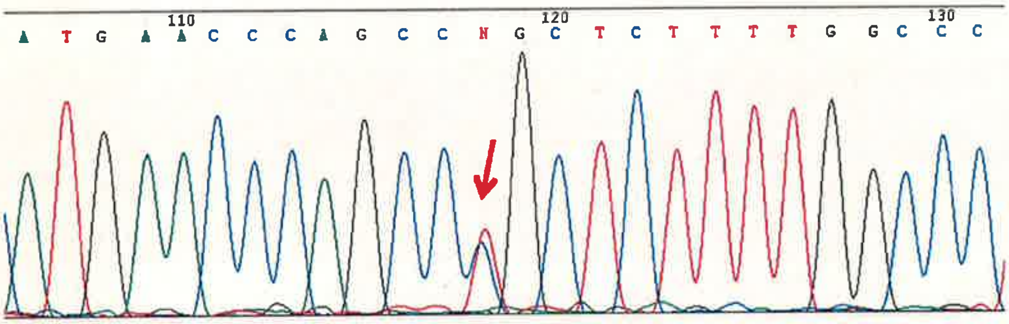

Translation of *AQP5* gene with the mutated residue circled in red:

```
  1 CCAGGCCGCCAGCCTCGGAGTGGGCGCGGGACAGTGCGCGGCGCCCCGCAGCCAGGCCCC
    ............................................................

 61 CGCCCCCGCCGCATCCACCTCCTCCGCCGCCTGCGACCCAACGGGCGCCCCCCGCCGCGG
    ............................................................

121 CAGCTGCCGCCGGGCCCCCGCGGCCACCATGAAGAAGGAGGTGTGCTCCGTGGCCTTCCT
    ............................-M--K--K--E--V--C--S--V--A--F--L

181 CAAGGCCGTGTTCGCAGAGTTCTTGGCCACCCTCATCTTCGTCTTCTTTGGCCTGGGCTC
 11 --K--A--V--F--A--E--F--L--A--T--L--I--F--V--F--F--G--L--G--S

241 GGCCCTCAAGTGGCCGTCGGCGCTGCCTACCATCCTGCAGATCGCGCTGGCGTTTGGCCT
 31 --A--L--K--W--P--S--A--L--P--T--I--L--Q--I--A--L--A--F--G--L
```

```
 301 GGCCATAGGCACGCTGGCCCAGGCCCTGGGACCCGTGAGCGGCGGCCACATCAACCCCGC
  51 --A--I--G--T--L--A--Q--A--L--G--P--V--S--G--G--H--I--N--P--A

 361 CATCACCCTGGCCCTCTTGGTGGGCAACCAGATCTCGCTGCTCCGGGCTTTCTTCTACGT
  71 --I--T--L--A--L--L--V--G--N--Q--I--S--L--L--R--A--F--F--Y--V

 421 GGCGGCCCAGCTGGTGGGCGCCATTGCCGGGGCTGGCATCCTCTACGGTGTGGCACCGCT
  91 --A--A--Q--L--V--G--A--I--A--G--A--G--I--L--Y--G--V--A--P--L

 481 CAATGCCCGGGGCAATCTGGCCGTCAACGCGCTCAACAACAACACAACGCAGGGCCAGGC
 111 --N--A--R--G--N--L--A--V--N--A--L--N--N--N--T--T--Q--G--Q--A

 541 CATGGTGGTGGAGCTGATTCTGACCTTCCAGCTGGCACTCTGCATCTTCGCCTCCACTGA
 131 --M--V--V--E--L--I--L--T--F--Q--L--A--L--C--I--F--A--S--T--D

 601 CTCCCGCCGCACCAGCCCTGTGGGCTCCCCAGCCCTGTCCATTGGCCTGTCTGTCACCCT
 151 --S--R--R--T--S--P--V--G--S--P--A--L--S--I--G--L--S--V--T--L

 661 GGGCCACCTTGTCGGAATCTACTTCACTGGCTGCTCCATGAACCCAGCCCGCTCTTTTGG
 171 --G--H--L--V--G--I--Y--F--T--G--C--S--M--N--P--A--R--S--F--G

 721 CCCTGCGGTGGTCATGAATCGGTTCAGCCCCGCTCACTGGGTTTTCTGGGTAGGGCCCAT
 191 --P--A--V--V--M--N--R--F--S--P--A--H--W--V--F--W--V--G--P--I

 781 CGTGGGGGCGGTCCTGGCTGCCATCCTTTACTTCTACCTGCTCTTCCCCAACTCCCTGAG
 211 --V--G--A--V--L--A--A--I--L--Y--F--Y--L--L--F--P--N--S--L--S

 841 CCTGAGTGAGCGTGTGGCCATCATCAAAGGCACGTATGAGCCTGACGAGGACTGGGAGGA
 231 --L--S--E--R--V--A--I--I--K--G--T--Y--E--P--D--E--D--W--E--E

 901 GCAGCGGGAAGAGCGGAAGAAGACCATGGAGCTGACCACCCGCTGACCAGTGTCAGGCAG
 251 --Q--R--E--E--R--K--K--T--M--E--L--T--T--R--*-..............

 961 GGGCCAGCCCCTCAGCCCCTGAGCCAAGGGGGAAAAGAAGAAAAAGTACCTAACACAAGC
     ............................................................

1021 TTCCTTTTTGCACAACCGGTCCTCTTGGCTGAGGAGGAGGAGCTGGTCACCCTGGCTGCA
     ............................................................

1081 CAGTTAGAGAGGGGAGAAGGAACCCATGATGGGACTCCTGGGGTAGGGGCCAGGGGCTGG
     ............................................................

1141 GGTCTGCTGGGGACAGGTCTCTCTGGGACAGACCTCAGAGATTGTGAATGCAGTGCCAAG
     ............................................................

1201 CTCACAGGCTGCAAGGGCCAGGCCAGAAAAGGGCGGGCCTGCAGCCTGCACCCCCCACCT
     ............................................................
```

```
1261 TCCCCAACCCTTCCTCAAGAGCTGAAGGGATCCCAGCCCCTAGGTGGGCAGAGGCAGACC
     ............................................................

1321 CTCCCCAGAGCTCCTTAGGAAGAACACAGACTGGTTCATTGAATGCCGCCTTATTTATTT
     ............................................................

1381 CTGGTGAGGATGCATGCGTGGGGCTGCTGGTGTTTAGAGTGGGGGCTACCCAATAAATCA
     ............................................................

1441 CTGATACTCA
     ..........
```

Key

Codons	Alternating codons	Alternating codons
Exons	Alternating exons	Alternating exons
Other features	UTR	

Variation summary

Variation	Genotype	Exon	Predicted change
c.562C>T	Heterozygous	3	p.Arg188Cys

Comments

The patient was heterozygous for the single nucleotide mutation c.562C>T in exon 3 of the *AQP5* gene, predicted to cause a missense change, p.Arg188Cys, at the protein level (Blaydon *et al.* (2013) *AJHG* **93**(2):330-5.).
